# *Gnetum chinense*, a new species of Gnetaceae from southwestern China

**DOI:** 10.3897/phytokeys.148.48510

**Published:** 2020-05-26

**Authors:** Wei-Yin Jin, Bing Liu, Shou-Zhou Zhang, Tao Wan, Chen Hou, Yong Yang

**Affiliations:** 1 State Key Laboratory of Systematic and Evolutionary Botany, Institute of Botany, Chinese Academy of Sciences, 20 Nanxincun, Xiangshan, Beijing 100093, China Institute of Botany, Chinese Academy of Sciences Beijing China; 2 Shenzhen Fairy Lake Botanical Garden, Shenzhen, China Tonghua Normal University Tonghua China; 3 Guangdong Provincial Key Laboratory of Silviculture, Protection and Utilization, Guangdong Academy of Forestry, Guangzhou, 510520, China; 4 Guangdong Academy of Forestry, Guangzhou, 510520, China Shenzhen Fairy Lake Botanical Garden Shenzhen China; 5 Tonghua Normal University, 950 Yucai Road, Dongchang District, Tonghua City, Jilin Province 134000, China Guangdong Academy of Forestry Guangzhou China

**Keywords:** *
Gnetum
*, China, morphology, phylogeny, taxonomy

## Abstract

*Gnetum chinense***sp. nov.**, a new lianoid species of Gnetaceae, is described from southwestern China. The new species is morphologically similar to *G. montanum* Markgr. in its oblong elliptic leaves and the ovoid to ellipsoid chlamydosperm, but differs from the latter by its shorter male spikes having fewer involucral collars (7–10 vs. 13–18 in *G. montanum*). We also did a new molecular analysis using one nuclear marker (i.e. nrITS) and four chloroplast markers (i.e. *mat*K gene, *rpo*C1 intron, *psb*B-*rps*12 IGS, and *trn*F-*trn*V IGS). The result suggests that this specific clade is sister to a large clade consisting of all other known Chinese lianoid species of *Gnetum* except *G. parvifolium* (Warb.) W.C. Cheng.

## Introduction

*Gnetum* L., belongs to the monotypic family Gnetaceae of gymnosperms, and contains ca. 40 extant species that are widely distributed in tropical and subtropical forests in Asia, Africa and South America (Yang et al. 2017a). This genus is evergreen, mostly lianas, rarely trees, and possesses a set of unusual characters for gymnosperms, e.g. dicots-like broad leaves with pinnate venation, female gametophytes lacking archegonia, male and female reproductive units assembled into whorls, male spikes usually having abortive chlamydosperms and appearing to be bisexual, chlamydosperms possessing two outer envelopes, etc. (Pearson 1929; Maheshwari and Vasil 1961; Martens 1971; Gifford and Foster 1989; Friedman and Carmichael 1996). Although the earliest macrofossil of the gnetoid clade is known from the mid-Jurassic in northeastern China, the modern *Gnetum* is believed to diversify in South America and split into the New World clade and the Old World clade around the K-Pg boundary (Hou et al. 2015; Yang et al. 2017b). The Chinese clade diverged from other southeastern Asian species around 38 mya (million years ago, 95% posterior density 27–49 mya), and became more diversified after the earliest Miocene (ca. 21 mya, Hou et al. 2016).

*Gnetum* has a wide range of distribution in southern China (Cheng 1978; Fu et al. 1999). Cheng (1978) recognized seven species in China, while Fu et al. (1999) accepted nine species in the *Flora of China*. Both studies were based on herbarium material only. These traditional taxonomic treatments laid much emphasis on reproductive characters, but variation patterns of important reproductive characters are ambiguous because i) fruiting material is poorly represented in herbaria, and ii) it is difficult to match male and female specimens to a certain species when studying a dioecious taxon like *Gnetum*.

Molecular phylogeny was successfully applied to the delimitation of species of *Gnetum* in combination with morphological characters (Hou et al. 2016; Kim and Won 2016). Kim and Won (2016) applied a barcode method and recognized three species in Cambodia, i.e. *G. macrostachyum* Hook. f., *G. montanum* Markgr., and G. aff. gracilipes C.Y. Cheng. Hou et al. (2016) conducted a taxonomic revision based on molecular and morphological data, and recognized the following six lianoid species in China: *G. catasphaericum* H. Shao, *G. formosum* Markgr., *G. luofuense* C.Y. Cheng, *G. montanum* Markgr., *G. parvifolium* (Warb.) W.C. Cheng, and *G. pendulum* C.Y. Cheng.

A new *Gnetum* species was identified when we worked on a *Gnetum* genome project a few years ago. Further morphological and molecular studies on newly collected materials during field investigations in southern China allowed us to describe this species here as new to science.

## Materials and methods

Plant materials, comprising silica-dried leaves and vouchers, were sampled in Yunnan and Guizhou of southern China. All vouchers were deposited in the Herbarium (PE), State Key Laboratory of Systematic and Evolutionary Botany, Institute of Botany, Chinese Academy of Sciences (Table 1).

**Table 1.** Sequences of *Gnetum chinense* sp. nov. generated in this study and their vouchers. All vouchers have been deposited in PE.

Collection	Locality	ITS	*mat*K	*rpoC*1	*psbB-rps*12	*trn*F-*trn*V
**T. Wan MLP001**	China. Yunnan: Malipo.	MT362085	MT373322	MT373311	MT373300	MT373333
**T. Wan MLP002**	China. Yunnan: Malipo		MT373323	MT373312	MT373301	MT373334
**T. Wan MLP003**	China. Yunnan: Malipo		MT373324	MT373313	MT373302	MT373335
**T. Wan MLP005**	China. Yunnan: Malipo		MT373325	MT373314	MT373303	MT373336
**B. Liu & al. 1360**	China. Yunnan: Malipo	MT362086	MT373326	MT373315	MT373304	MT373337
**B. Liu & al. 1441**	China. Yunnan: Malipo		MT373327	MT373316	MT373305	MT373338
**B. Liu & al. 1725**	China. Yunnan: Malipo		MT373328	MT373317	MT373306	MT373339
**B. Liu & al. 2627**	China. Yunnan: Cangyuan	MT362087	MT373329	MT373318	MT373307	MT373340
**B. Liu & al. 2675**	China. Yunnan: Lancang	MT362088	MT373330	MT373319	MT373308	MT373341
**B. Liu & al. 3045**	China. Yunnan: Jiangcheng	MT362089	MT373331	MT373320	MT373309	MT373342
**C.Y. Deng 12466**	China. Guizhou: Xingyi	MT362084	MT373321	MT373310	MT373299	MT373332

Total genomic DNA was extracted from the dried leaf materials using the CTAB method (Doyle and Doyle 1987) and purified using a QIAquick PCR Purification Kit. For phylogenetic studies, it is thought that nrITS and four chloroplast markers including *mat*K gene, *rpo*C1 intron, *psb*B-*rps*12 IGS, and *trn*F-*trn*V IGS are highly variable and suitable for delimiting species of *Gnetum* (Kim and Won 2016; Hou et al. 2016). We followed the methods described in Hou et al. (2016), and one nuclear marker (i.e. nrITS) and four chloroplast markers (i.e. *mat*K gene, *rpo*C1 intron, *psb*B-*rps*12 IGS, and *trn*F-*trn*V IGS) were targeted. *Gnetum* sequences generated in Hou et al. (2016) were downloaded from the GeneBank (Table 2). Sanger sequencing was conducted at Majorbio, Beijing, China. The output files were assembled and edited using Sequencer ver. 4.5 (Gene Codes Corporation, Ann Arbor, Michigan, U.S.A.) and DNA sequences were aligned using Clustal X ver. 2.1 (Larkin et al. 2007) and manually adjusted using BioEdit ver. 7.2.5 (Hall 1999). Sequences of the five markers were concatenated using SequenceMatrix Windows ver. 1.7.8 (Vaidya et al. 2011).

**Table 2.** *Gnetum* sequences obtained from the GenBank for this study.

Species	ITS	*mat*K	*rpo*C1	*psb*B-*rps*12	*trn*F-*trn*V
***G. catasphaericum*_24**	KX234206	KX234250	KX234304	KX234352	KX234382
***G. catasphaericum*_31**	KX234205	KX234249	KX234303	KX234351	KX234381
***G. catasphaericum*_32**	KX234203	KX234247	KX234301	KX234349	KX234379
***G. catasphaericum*_44**	KX234202	KX234246	KX234300	KX234348	–
***G. cataspharicum*_33**	KX234204	KX234248	KX234302	KX234350	KX234380
***G. cuspidatum*_20**	KX234174	KX234222	KX234274	KX234325	–
***G. cuspidatum*_C52**	KP256660	KP256698	KX234273	KX234324	–
***G. diminutum*_C101**	KP256664	KP256702	KX234275	–	–
***G. edule*_C4**	KP256658	KP256696	KX234268	KX234322	–
***G. formosum*_14**	KX234200	KX234230	KX234298	KX234346	KX234377
***G. formosum*_21**	KX234201	KX234231	KX234299	KX234347	KX234378
***G. giganteum*_37**	KX234209	KX234252	KX234307	KX234354	KX234384
***G. giganteum*_41**	KX234213	KX234256	KX234311	KX234358	KX234388
***G. giganteum*_8**	KX234211	KX234254	KX234309	KX234356	KX234386
**G. gnemon var. brunonianum_1**	KX234173	KX234221	KX234265	KX234319	–
**G. gnemon var. brunonianum_102**	–	KX385188	KX385188	KX385188	KX385188
**G. gnemon var. gnemon_2**	KX234170	KX234219	KX234263	KX234317	–
**G. gnemon var. gnemon_101**	KX234171	KX385189	KX385189	KX385189	KX385189
**G. gnemon var. griffithii_23**	KX234172	KX234220	KX234264	KX234318	–
***G. gnemonoides*_C51**	KP256656	KP256694	KX234266	KX234320	–
***G. gracilipes*_39**	KX234210	KX234253	KX234308	KX234355	KX234385
***G. gracilipes*_42**	KX234214	KX234257	KX234312	KX234359	–
***G. haianense*_15**	KX234184	KX234232	KX234283	KX234333	KX234364
***G. hainanense*_10**	KX234195	KX234241	KX234293	KX234341	KX234373
***G. hainanense*_11**	KX234193	KX234238	KX234290	KX234338	KX234370
***G. hainanense*_12**	KX234194	KX234239	KX234291	KX234339	KX234371
***G. hainanense*_13**	KX234198	KX234244	KX234296	KX234344	KX234375
***G. hainanense*_16**	KX234199	KX234245	KX234297	KX234345	KX234376
***G. hainanense*_18**	KX234196	KX234242	KX234294	KX234342	–
***G. hainanense*_25**	–	KX234240	KX234292	KX234340	KX234372
***G. hainanense*_5**	KX234197	KX234243	KX234295	KX234343	KX234374
***G_hainanense*_9**	KX234192	KX234237	KX234289	KX234337	KX234369
***G_hainanense*_107**	KX234187	KX385193	KX385193	KX385193	KX385193
***G. hainanense*_110**	KX234186	KX385194	KX385194	KX385194	KX385194
***G. latifolium*_C15**	KP256661	–	KX234269	–	–
***G. leptostachyum*_C102**	KP256665	KP256703	KX234271	–	–
***G. luofuense*_19**	KX234190	KX234236	KX234287	KX234336	KX234367
***G. luofuense*_27**	KX234189	KX234235	KX234286	–	–
***G. luofuense*_28**	KX234185	KX234233	KX234284	KX234334	KX234365
***G. luofuense*_6**	KX234188	KX234234	KX234285	KX234335	KX234366
***G. luofuense*_C61**	KX234191	KP256710	KX234288	–	KX234368
***G. montanum*_105**	–	KX385195	KX385195	KX385195	KX385195
***G. montanum*_106**	–	KX385196	KX385196	KX385196	KX385196
***G. montanum*_17**	KX234208	KX234251	KX234306	KX234353	KX234383
***G. montanum*_29**	KX234207	–	KX234305	–	–
***G. neglectum*_C19**	KP256667	KP256705	KX234270	–	–
***G. parvifoilum*_35**	KX234178	KX234224	KX234277	KX234327	–
***G. parvifolium*_108**	KX234176	KX385190	KX385190	KX385190	KX385190
***G. parvifolium*_109**	KX234177	KX385192	KX385192	KX385192	KX385192
***G. parvifolium*_22**	KX234181	KX234227	KX234280	KX234330	–
***G. parvifolium*_26**	KX234180	KX234226	KX234279	KX234329	–
***G. parvifolium*_34**	KX234179	KX234225	KX234278	KX234328	–
***G. parvifolium*_36**	KX234175	KX234223	KX234276	KX234326	–
***G. parvifolium*_40**	KX234182	KX234228	KX234281	KX234331	–
***G. parvifolium*_43**	KX234183	KX234229	KX234282	KX234332	–
***G. parvifolium*_C50**	KP256675	KX385191	KX385191	KX385191	KX385191
***G. pendulum*_103**	–	KX385197	KX385197	KX385197	KX385197
***G. pendulum*_104**	–	KX385198	KX385198	KX385198	KX385198
***G. pendulum*_38**	KX234212	KX234255	KX234310	KX234357	KX234387
***G. pendulum*_4**	KX234217	KX234260	KX234315	KX234362	KX234391
***G. pendulum*_45**	KX234216	KX234259	KX234314	KX234361	KX234390
***G. pendulum*_46**	KX234215	KX234258	KX234313	KX234360	KX234389
***G. pendulum*_47**	KX234218	KX234261	KX234316	KX234363	–
***G. raya*_C11**	KP256657	–	KX234267	KX234321	–
***G. tenuifolium*_C18**	KP256662	KP256700	KX234272	KX234323	–
***G. africanum*_C41**	KP256642	KP256681	KX234262	–	–

Previous studies suggested that the African species are sister to all Asian species (Won and Renner 2006; Hou et al. 2015), as a result, we chose the African *G. africanum* Welw. as the outgroup. Maximum likelihood (ML) analyses were conducted using the RAxM L-HPC2 on XSEDE (8.0.0) executed in the CIPRES portal (http://www.phylo.org/, Stamatakis 2014). The ML bootstrap values (BS) for each node were summarized after 1,000 replicates of bootstrapping iterations. The obtained trees were viewed and edited using FigTree ver. 1.4.0 (http://tree.bio.ed.ac.uk/software/figtree/). Bayesian inference (BI) analyses were performed using MrBayes 3.2.6 (Huelsenbeck and Ronquist 2001) on XSEDE (8.0.0) in CIPRES. The Markov Chain Monte Carlo (MCMC) algorithm was run for 3,000,000 generations with the sampling frequency 1,000. Bayesian posterior probabilities (PP) were calculated for the majority consensus tree of all sampled trees after discarding trees sampled within the burn-in (25%) phase in MrBayes v.3.2.1.

The distribution map was generated using ArcGIS 9.3 (ESRI, Redlands, CA, USA; http://www.esri.com). The photos were taken using digital cameras (Nikon D700 and Olympus TG-3), manually edited and created using Adobe Photoshop CS2 ver. 9.0. Phylogenetic trees were viewed and adjusted using FigTree ver. 1.4.0 (http://tree.bio.ed.ac.uk/software/figtree/).

## Results

### Phylogeny

**Figure 1.**
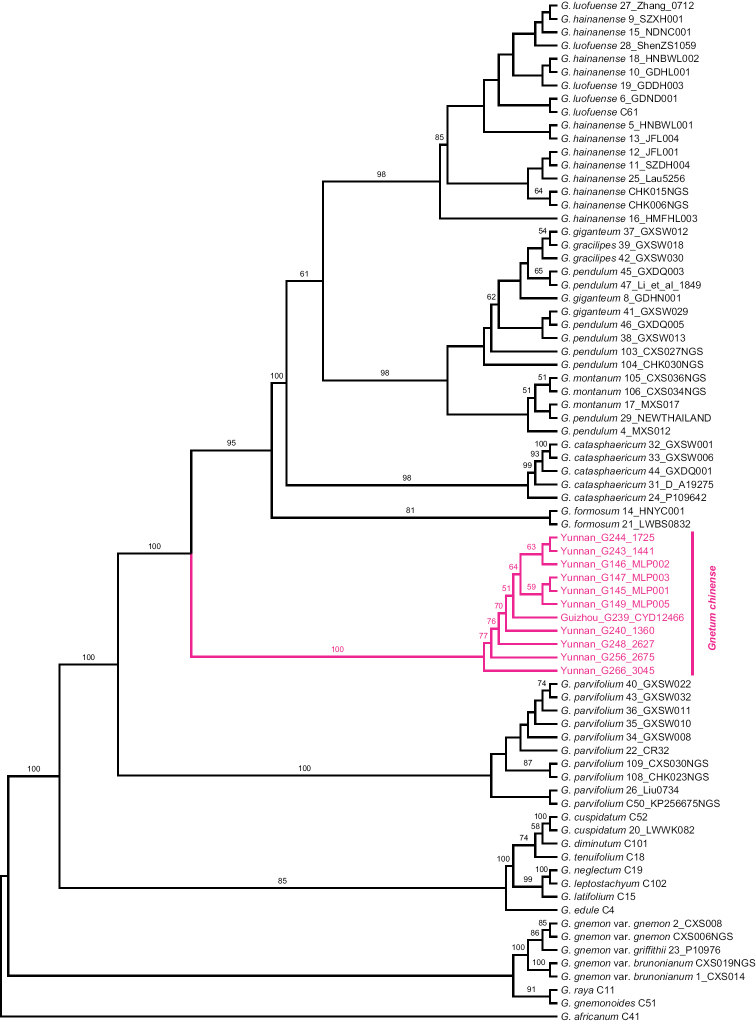
Maximum likelihood tree based on nuclear ribosomal ITS and chloroplast *mat*K, *rpo*C1, *psb*B-*rps*12, and *trn*F-*trn*V, showing the robust species clade of *Gnetum chinense* sp. nov. Bootstrap values are displayed when they are greater than 50%.

**Figure 2.**
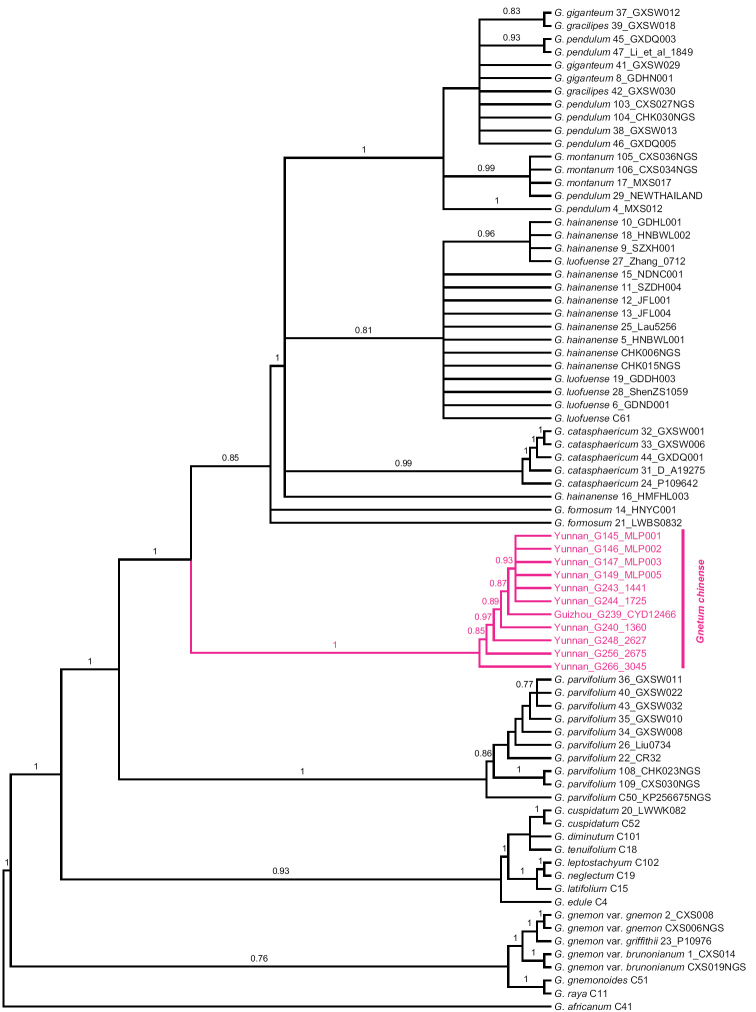
Bayesian inference tree based on nuclear ribosomal ITS and chloroplast *mat*K, *rpo*C1, *psb*B-*rps*12, and *trn*F-*trn*V, showing the robust species clade of *Gnetum chinense* sp. nov. Posterior probabilities are shown when they are greater than 0.70.

The ML tree (Fig. 1), in general, was better resolved than the BI tree (Fig. 2). All Chinese lianoid taxa of *Gnetum* included in this study formed a strongly supported monophyletic group (BS: 100%; PP: 1.00). Ten samples of *G. parvifolium* constituted a sister group (BS: 100%; PP: 1.00) to a clade consisting of the rest of the lianoid congeners from China included in this study. The analyses revealed the 11 newly collected specimens as a monophyletic group (BS: 100%; PP: 1.00) sister to a clade composed of *G. formosum*, *G. catasphaericum*, *G. luofuense*, *G. montanum* and *G. pendulum*. Delimitations between *G. montanum* and *G. pendulum* were not resolved. The two samples of *G. formosum* formed a weakly supported group (BS: 81%; PP < 0.70), which was followed by a split between the strongly supported *G. catasphaericum* (BS: 98%; PP: 0.99) and a large clade containing a subclade of *Gnetum montanum*, *G. pendulum*, *G. giganteum* H. Shao, and *G. gracilipes* (BS: 98%; PP: 1.00), and another one of *G. hainanense* C.Y. Cheng ex L.K. Fu et al. and *G. luofuense* (BS: 98%; PP: 0.81). Delimitations between the two species were not resolved.

### Taxonomy

#### Gnetum chinense

Taxon classificationPlantaeGnetalesGnetaceae

Y. Yang, Bing Liu & S.Z. Zhang sp. nov.

77DD1B45-1A13-5730-AD08-D0752659A11F

urn:lsid:ipni.org:names:77209708-1

Figs 3 , 4

##### Type.

China. Yunnan: Cang-yuan County, on the way from Ban-hong to Ban-lao Prefecture, forest margin, male cones, March 31^st^, 2015, B. Liu, Y. Yang & T.W. Xiao 2627 (PE, holotype).

**Figure 3.**
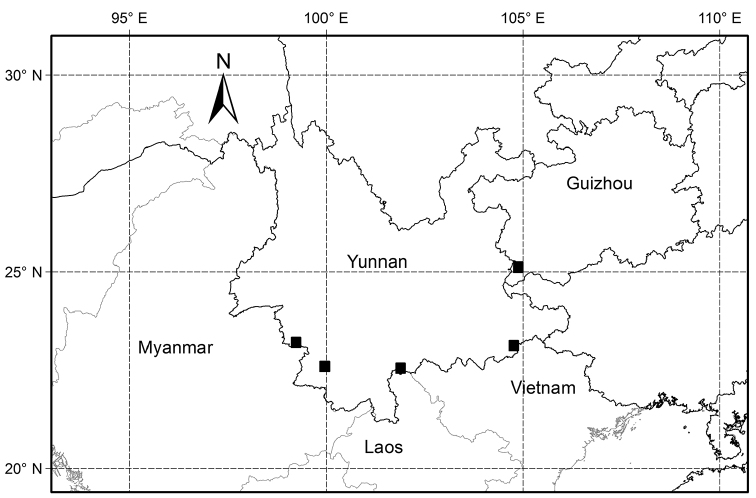
Map displaying the distribution of *Gnetum chinense* sp. nov. (black squares).

##### Diagnosis.

This species is similar to *G. montanum* in its oblong elliptic leaves and subsessile chlamydosperm, but differs from the latter by its shorter male cones (1–1.5 cm long in the new species vs. 2–3 cm in *G. montanum*) having fewer involucral collars (7–10 in the new species vs. 13–18 in *G. montanum*), nearly sessile or extremely shortly stiped chlamydosperms (vs. markedly stiped, stipes 3–5 mm long in *G. montanum*).

##### Description.

**Figure 4.**
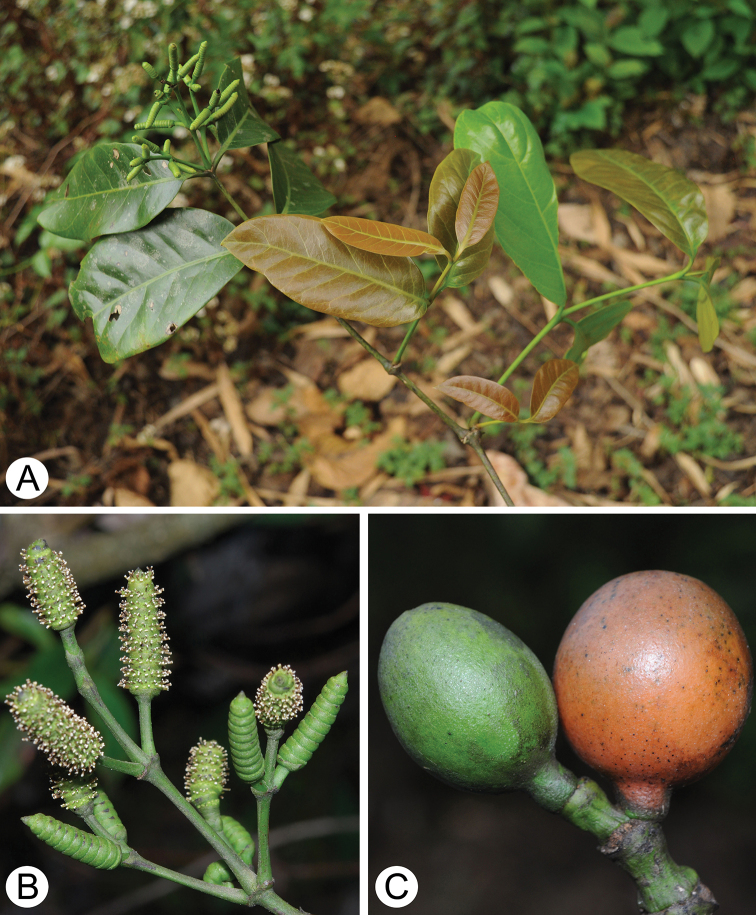
*Gnetum chinense* sp. nov. **A** branch bearing male cones **B** male cones **C** female cone portion displaying chlamydosperm morphology.

Lianas; twigs terete, dichasially branched having swollen nodes. Leaves opposite (Fig. 4a), oblong to elliptic, 11–16 cm long, 4–8 cm wide, base rotund to acute, apex acute to acuminate, pinnately veined, midvein impressed adaxially and elevated abaxially, lateral veins 6–8 (Fig. 4a), more or less elevated on both sides, petioles 1–1.2 cm long, grooved adaxially. Male reproductive shoots terminal, dichasial, branched once or twice (Fig. 4a). Male cones pedunculate, peduncles 2–10 mm long; cylindric, ca. 10–15 mm long, 4 mm in diam., involucral collars 8–10 (Fig. 4b). Chlamydosperms ellipsoid to subglobose, ca. 2.2 cm long, 1.4 cm in diam., apex obtuse, base contracted into an extremely short stalk or subsessile, green when young, and orange when mature (Fig. 4c).

##### Distribution.

In Yunnan and Guizhou provinces of China (Fig. 3).

##### Habitats.

In evergreen tropical and subtropical forests.

##### Etymology.

The specific epithet ‘*chinense*’ is derived from China.

##### Phenology.

Blooming male cones and mature chlamydosperms were found in late May and early November, respectively.

##### Conservation.

*Gnetum chinense* is common in evergreen forests in Yunnan and Guizhou. We consider this species to be of Least Concern (LC) under the IUCN Red List Categories and Criteria ver. 3.1 second edition (IUCN 2012).

##### Specimens examined.

**China.** Yunnan: Lan-cang Lahuzu Autonomous County, from Shang-yun to Xi-meng, Apr. 2, 2015, *B. Liu, Y. Yang & T.W. Xiao 2675* (PE); Jiang-cheng County, Qu-shui Prefecture, Apr. 18, 2015, *B. Liu 3045* (PE); Ma-li-po County, March 15–17, 2015, *T. Wan MLP001, MLP002, MLP003, MLP 005* (PE); Ma-li-po County, Xia-jin-chang Prefecture, Li-jia-wan, May 27, 2011, *B. Liu 1360* (PE); Ma-li-po County, Xia-jin-chang Prefecture, Li-jia-wan, Sept. 24, 2011, *B. Liu 1441* (PE); Ma-li-po County, Xia-jin-chang Prefecture, Li-jia-wan, Nov. 2, 2012, *B. Liu 1725* (PE). Guizhou: unknown collection date, *C.Y. Deng CYD12466* (PE).

## Discussion

Phylogenies based on molecular data have clearly resolved major lineages of *Gnetum*, including a South American clade, an African clade, and several Asian clades (Won and Renner 2003, 2005a, 2005b, 2006; Kim and Won 2016; Hou et al. 2015, 2016). Taxonomy of the Asian *Gnetum* is rather complicated because plants of the genus are usually dioecious woody climbers, and there are few taxonomic characters, so it is difficult to identify species without diagnostic reproductive characters (Kim and Won 2016).

Phylogenetic methods were successfully applied to discover and delimit species of Asian *Gnetum* (Kim and Won 2016; Hou et al. 2016). Our phylogenetic study found a new specific clade that was not recognized in previous studies; this clade is well resolved (Figs 1, 2; BS: 100%; PP: 1.00).

We did a morphological comparison between our new species and those known lianoid species from China (Table 3), and found that the specimens of this new specific clade are similar to *G. montanum* in the shape of leaves and chlamydosperms, and to *G. parvifolium* in the length of the male spikes and number of involucral collars, but differ from *G. montanum* by their shorter male cones having fewer involucral whorls, and from *G. parvifolium* by their larger leaves 11–16 cm long and bigger chlamydosperms ca. 2.2 cm long (vs. smaller leaves ca. 4–11 cm long, smaller chlamydosperms 1.3–1.8 cm long).

**Table 3.** A morphological comparison between *Gnetum chinense* and other Chinese lianoid species.

Species	Leaf blade shape	Leaf blade length (cm)	Leaf blade width (cm)	Petiole length (mm)	Male spike length (cm)	Male spike involucral collars	Chlamydosperm shape	Chlamydosperm length (cm)	Chlamydosperm stipe length (mm)
***Gnetum catasphaericum* H. Shao**	Ovate to oblong ovate	7–12	4–6.5	6–10	ca. 2	10–16	Oblong, subglobose	1.8–2.2	2–6
***G. chinense* sp. nov.**	Oblong to elliptic	11–16	4–8	10–12	1–1.5	8–10	Ellipsoid to subglobose	ca. 2.2	Subsessile
***G. formosum* Markgr.**	Elliptic to narrowly oblong	11–14	4–7	9–10	?	?	Narrowly oblong, fusiform	2–2.5	Sessile
***G. luofuense* C.Y. Cheng**	Elliptic to oblong ovate	4.5–16	3–8.5	8–13	2–3	12–15	Broadly ellipsoid to cylindric	1.8–2.5	2–5
***G. montanum* Markgr.**	Elliptic to oblong	10–28	4.5–13	9–26	2–3.5	16–25	Cylindric ovoid, cylindric	1.6–2	3–5
***G. parvifolium* (Warb.) W.C. Cheng**	Elliptic to narrowly oblong	4–11	2–4	5–7	0.8–1.5	9–11	ellipsoid	1.3–1.8	Sessile
***G. pendulum* C.Y. Cheng**	Narrowly elliptic to oblong ovate	10–18	4–8.5	8–15	1–1.5	12–15	Elongate ellipsoid	3–4	10–30

Data were collected from Hou et al. (2016) and this study.

A few morphological details of the new species are taxonomically important but not known to us, e.g. shape and the number of sterile ovules in male spike. As a result, further field investigations are encouraged.

## Supplementary Material

XML Treatment for Gnetum chinense
